# Sly-miR398 Participates in Cadmium Stress Acclimation by Regulating Antioxidant System and Cadmium Transport in Tomato (*Solanum lycopersicum*)

**DOI:** 10.3390/ijms24031953

**Published:** 2023-01-19

**Authors:** Guochao Yan, Yuchen Hua, Han Jin, Qingying Huang, Guanfeng Zhou, Yunmin Xu, Yong He, Zhujun Zhu

**Affiliations:** Key Laboratory of Quality and Safety Control for Subtropical Fruit and Vegetable, Ministry of Agriculture and Rural Affairs, Collaborative Innovation Center for Efficient and Green Production of Agriculture in Mountainous Areas of Zhejiang Province, College of Horticulture Science, Zhejiang A&F University, Hangzhou 311300, China

**Keywords:** miR398, cadmium (Cd), tomato, antioxidant system, Cd uptake and translocation

## Abstract

Cadmium (Cd) pollution is one of the major threats in agricultural production, and can cause oxidative damage and growth limitation in plants. MicroRNA398 (miR398) is involved in plant resistance to different stresses, and the post-transcriptional regulation of miR398 on *CSD*s plays a key role. Here, we report that miR398 was down-regulated in tomato in response to Cd stress. Simultaneously, *CSD1* and SOD were up-regulated, with *CSD2* unchanged, suggesting *CSD1* is involved in miR398-induced regulation under Cd stress. In addition, the role of miR398 in Cd tolerance in tomato was evaluated using a transgenic line overexpressing *MIR398* (miR398#OE) in which the down-expression of miR398 was disrupted. The results showed that Cd stress induced more significant growth inhibition, oxidative damage, and antioxidant enzymes disorder in miR398#OE than that in wild type (WT). Moreover, higher Cd concentration in the shoot and xylem sap, and net Cd influx rate, were observed in miR398#OE, which could be due to the increased Cd uptake genes (*IRT1*, *IRT2*, and *NRAMP2*) and decreased Cd compartmentalization gene *HMA3*. Overall, our results indicate that down-regulated miR398 plays a protective role in tomato against Cd stress by modulating the activity of antioxidant enzymes and Cd uptake and translocation.

## 1. Introduction

Cadmium (Cd) is one of the major metal pollutants in arable soil, and adversely affects plant growth and food safety [1]. With an increase in chemical fertilizer application and sewage irrigation in agricultural practice, the area of arable lands under Cd pollution is growing larger, thereby threatening sustainable agricultural production severely [2,3]. Cd stress causes numerous toxic effects including membrane lipid peroxidation, photosynthesis system destruction, and growth limitation in plants, which is, at least partially, due to Cd-induced accumulation of reactive oxygen species (ROS) [4,5], since the ability of ROS scavenging in plants is disrupted by Cd stress [6,7]. Superoxide dismutase (SOD) plays an important role as the first line of antioxidant defense in plants to convert superoxide anion (O_2_^−^) into H_2_O_2_, thereby participating in ROS scavenging and stress acclimation in plants [8,9]. Considering the role of SOD in antioxidant defense and Cd-induced oxidative toxicity, it is not surprising that the connection between SOD and Cd stress tolerance has been repeatedly documented in different kinds of plants species including rice [10], wheat [11], and tomato [12,13].

MicroRNAs (miRNAs) play diverse roles in plant development and stress response by regulating gene expression through directing mRNA cleavage and/or inhibiting translation [14]. As a key regulator of SOD in plants, microRNA398 (miR398) can negatively regulate the expression of *CSD*s (encoding Cu-Zn SOD), thereby modulating the activity of SOD and participating in ROS scavenging [15]. After the first report of miR398 in *Arabidopsis* [16], miR398 has been identified in different plants including melon [17], grape [18], and tomato [19,20]. Due to the pivotal role of SOD in antioxidant defense under stress conditions, miR398 has been found to participate in the response to kinds of abiotic and biotic stresses in plants including salt stress, drought stress, heat stress, metal stress, and pathogen diseases [21,22,23]. Under metal stress including copper, nickel, and cadmium, miR398 has been shown to be involved in metal stress tolerance in different plant species including *Arabidopsis* [24,25], alfalfa [26], caster bean [27], and grapevine [28].

However, it should be noted that the expression of miR398 differs among stress types and plant species, which leads to distinct regulation on SOD and antioxidant defense [21]. In *Arabidopsis*, salt stress, cold stress, and high light reduced the expression of miR398 [16,25,29], while up-regulated expression of miR398 was observed under heat stress [30] and UV-B radiation [31]. In addition, drought stress promoted the expression of miR398 in wheat [32], while it down-regulated the expression of miR398 in rice [33]. Moreover, although both *CSD1* and *CSD2* are target genes of miR398, the regulative effects of miR398 on these two *CSD*s to miR398 could be different in miR398-induced modulation of SOD activity in plants under stress conditions. For example, the down-regulated expression of miR398 promoted the expression of both *CSD1* and *CSD2* under light stress [25], but enhanced the expression of *CSD1* but not *CSD2* under salt stress [29] in *Arabidopsis*. However, the response of miR398 to Cd stress and the regulative effects of miR398 on *CSD*s and SOD under Cd exposure are still largely unknown.

In plants under stressful conditions, the collaboration of different antioxidant enzymes plays the critical role in scavenging excess ROS and maintaining redox homeostasis, while the antioxidant enzymes could affect each other in the sophisticated antioxidant system [34]. Therefore, the regulation effects of miR398 on *CSD*s and SOD could successively influence the activity of other antioxidant enzymes. For example, in tomato under salt stress, overexpression of *MIR398* regulated the activity of APX and CAT in addition to SOD [35]. Lin et al. [36] indicated that miR398-regulated antioxidants were involved in the accumulation of mosaic virus in bamboo, while the results of Li et al. [37] showed that miR398 could regulate the activity of SOD and CAT, thereby modulating ROS production and blast disease resistance in rice. In addition, under the stresses induced by toxic elements (e.g., salt stress, metal stress), membrane peroxidation caused by ROS imbalance would influence the trans-membrane transport of toxic materials [38], implying that miR398-regulated antioxidant defense could affect the uptake and transport of toxic materials such as Cd, although the hypothesis remains untested.

Tomato (*Solanum lycopersicum*) is an important horticultural crop worldwide. In this study, the response of miR398 to Cd exposure in tomato seedlings was investigated. In addition, a transgenic line of tomato overexpressing *MIR398* (miR398#OE) in which the down-regulation miR398 in response to Cd was disrupted was used in this study. Tomato growth parameters, oxidative damage, ROS accumulation, antioxidant enzyme activity, Cd concentration, and expression of genes concerning Cd uptake and translocation were determined and compared in this study. The results showed that the down-expression of miR398 played a protective role against Cd stress in tomato by modulating antioxidant enzymes and Cd uptake and translocation.

## 2. Results

### 2.1. Down-Regulated MiR398 Promoted CSD1 and SOD in Response to Cd Toxicity

**Figure 1:**
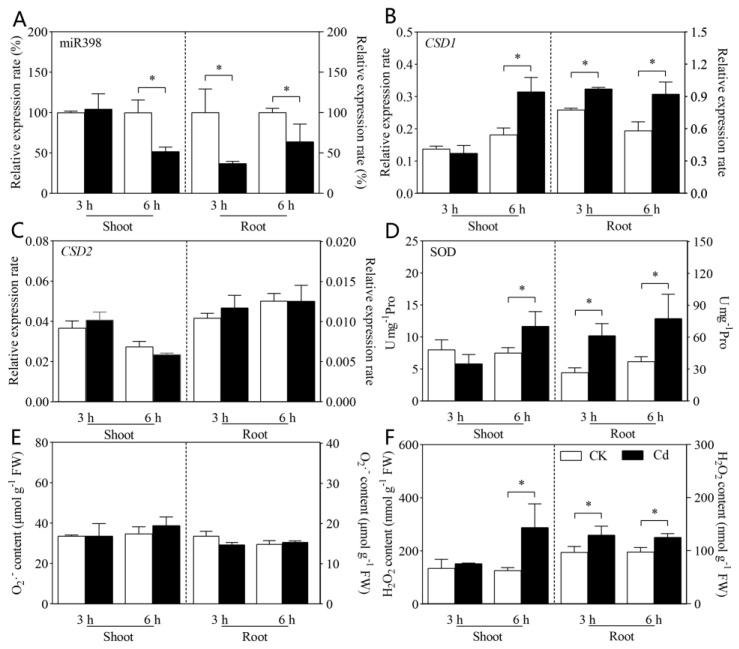
Effects of cadmium stress on miR398 (**A**), *CSD1* (**B**), *CSD2* (**C**), SOD (**D**), O_2_^−^ (**E**), and H_2_O_2_ (**F**) in tomato shoot and root. Tomato seedlings (25 days old, cv. Microtom) were treated with control (CK) and 100 μM Cd(NO_3_)_2_ (Cd) for 3 or 6 h. The data are the mean values + SD of four individual replicates and asterisks indicate significant differences between CK and Cd treatment (*p* < 0.05). One unit (U) of activity of SOD is defined as 50% inhibition of the photochemical reduction of nitroblue tetrazolium. SOD, superoxide dismutase, Pro, protein.

At the outset, the expression of miR398 in tomato shoot and root was measured at 3 and 6 h after treatment (HAT) of 100 μM Cd(NO_3_)_2_. The expression of miR398 was down-regulated in both the shoot and root of tomato under Cd stress (Figure 1A), while the down-regulated expression of miR398 in response to Cd stress was earlier in the root than that in the shoot. In tomato root, the expression of miR398 was significantly reduced by Cd stress at 3 HAT, while the expression of miR398 in the shoot was inhibited under Cd exposure at 6 HAT (Figure 1A).

Along with the down-expression of miR398, the expression of *CSD1* and the activity of SOD was promoted in both the shoot and root under Cd stress (Figure 1B,D), while no obvious change in *CSD2* expression was observed (Figure 1C). As for reactive oxygen species (ROS) concentration, although no obvious change in O_2_^−^ content was induced by Cd stress (Figure 1E), the content of H_2_O_2_ was significantly increased in both the shoot and root of tomato under Cd exposure (Figure 1F). In accordance with the time-dependent response of miR398 to Cd stress, an earlier increase in *CSD1*, SOD, and H_2_O_2_ was also observed in the root (at 3 HAT) than that in the shoot (at 6 HAT) of tomato under 100 μM Cd treatment (Figure 1).

### 2.2. Cd Stress Induced More Significant Growth Inhibition and Oxidative Damage in MiR398#OE

**Figure 2:**
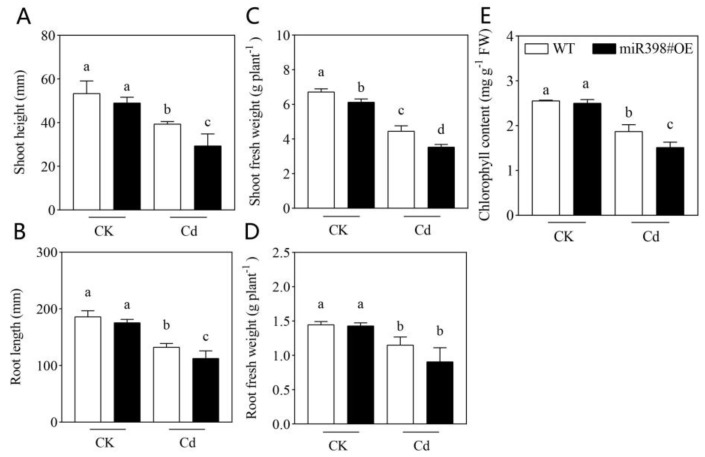
Effects of cadmium stress on tomato growth in wild type (WT) and transgenic line overexpressing *MIR398* (miR398#OE). Shoot height (**A**), root length (**B**), fresh weight of the shoot (**C**) and root (**D**), and chlorophyll content (**E**). Tomato seedlings (25 d old) of WT and miR398 #OE were grown under control (CK) and 100 μM Cd(NO_3_)_2_ (Cd) for 9 d. The data are the mean values + SD of four individual replicates and different letters indicate significant differences (*p* < 0.05). FW, fresh weight.

Based on the results mentioned above, a transgenic line overexpressing *MIR398* (miR398#OE) in which the down-regulation of miR398 was disrupted was used in this study, and growth parameters including shoot height, root length, biomass, and chlorophyll content were determined and compared in wild type (WT, cv. Microtom) and miR398#OE after 9 d of treatment. As shown in Figure 2, no significant difference in growth was found between WT and miR398#OE under control, while Cd stress caused severe growth inhibition in both WT and miR398#OE. However, more significant decline in shoot height, root length, shoot biomass, and chlorophyll content was observed in miR398#OE under Cd treatment in contrast with WT (Figure 2).

**Figure 3:**
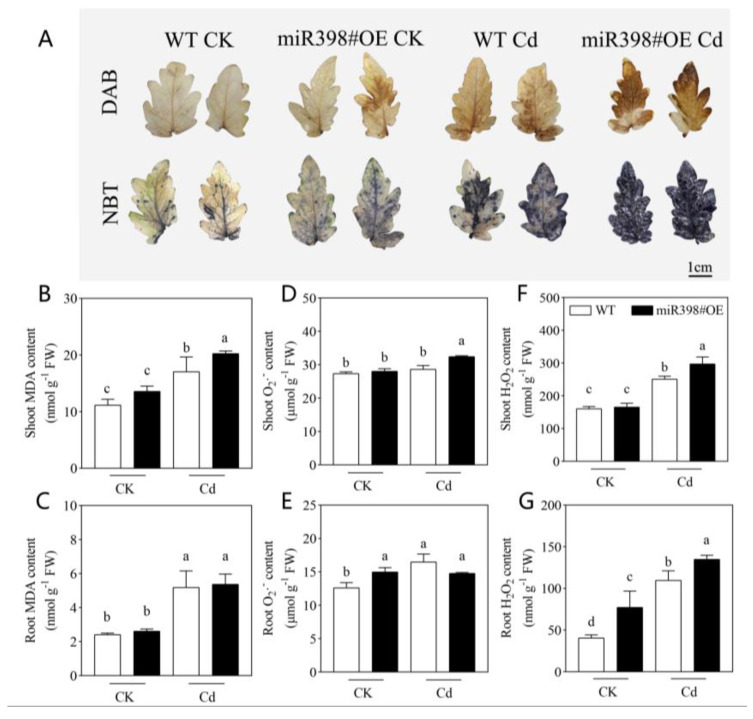
Cadmium stress-induced oxidative damage in wild type (WT) and transgenic line overexpressing *MIR398* (miR398#OE). Superoxide anion (O_2_^−^) and H_2_O_2_ visualization using nitroblue tetrazolium (NBT) and diaminobenzidine (DAB) staining (**A**), malonaldehyde (MDA) content in the shoot (**B**) and root (**C**), O_2_^−^ content in the shoot (**D**) and root (**E**), and H_2_O_2_ content in the shoot (**F**) and root (**G**). Tomato seedlings (25 d old) of WT and miR398#OE were grown under control (CK) and 100 μM Cd(NO_3_)_2_ (Cd) for 9 d. The data are the mean values + SD of four individual replicates and different letters indicate significant differences (*p* < 0.05). FW, fresh weight.

To determine oxidative damage induced by Cd stress in tomato, nitroblue tetrazolium (NBT) and diaminobenzidine (DAB) staining was used for superoxide anion (O_2_^−^) and H_2_O_2_ visualization, and the contents of malonaldehyde (MDA), H_2_O_2_, and O_2_^−^ in tomato shoot and root were measured. Under the control condition, overexpression of *MIR398* caused no significant change in MDA, H_2_O_2_, and O_2_^−^ in the shoot, while increased contents of H_2_O_2_ and O_2_^−^ were found in miR398#OE root, with MDA unchanged in comparison with WT (Figure 3). In addition, severe oxidative damage was induced by Cd treatment in both WT and miR398#OE (Figure 3). However, the results of NBT and DAB staining showed that 100 μM Cd stress induced more significant accumulation of O_2_^−^ and H_2_O_2_ in tomato leaf of miR398#OE than that in WT (Figure 3A). Consistently, the contents of MDA, H_2_O_2_, and O_2_^−^ in the shoot were more significantly increased by Cd stress in miR398#OE in contrast with WT (Figure 3). In tomato root, Cd stress induced more significant accumulation of H_2_O_2_ in miR398#OE, but no obvious change in MDA and O_2_^−^ was found between WT and miR398#OE under Cd exposure (Figure 3).

### 2.3. Overexpression of MIR398 Enhanced Cd-Induced Disorder of Antioxidant Enzymes in Tomato

Under control treatment, overexpression of *MIR398* decreased the activity of SOD and APX, with CAT, GPOD, GR, and DHAR unchanged in tomato shoot. In the root, overexpression of *MIR398* decreased the activity of SOD, CAT, and GR and increased the activity of APX, with no effect on GPOD and DHAR (Table 1).

As for the effects of Cd stress on antioxidant enzymes, 100 μM Cd induced significant changes in the activity of antioxidant enzymes in both the shoot and root of WT (Table 1). In tomato shoot, Cd stress decreased the activity of SOD, CAT, and APX, and increased the activity of GPOD and DHAR in WT (Table 1). Under Cd exposure, more significant decrease in SOD, CAT, APX, and increase in GPOD was observed in the shoot of miR398#OE in comparison with that in WT (Table 1). However, the aggregative effects of *MIR398* overexpression on Cd-induced antioxidant enzyme disorder in tomato shoot was not found in tomato root. Although Cd stress increased the activity of APX, GR, and DHAR and decreased the activity of SOD in the root of WT, no obvious difference in the activity of these enzymes was found between WT and miR398#OE under Cd exposure (Table 1). In addition, Cd stress increased the activity of GPOD in the root of WT with no effect on CAT, while overexpression of *MIR398* decreased the activity of CAT and GPOD under Cd stress (Table 1).

### 2.4. Overexpression of MIR398 Enhanced Cd Uptake and Accumulation in Tomato

**Figure 4:**
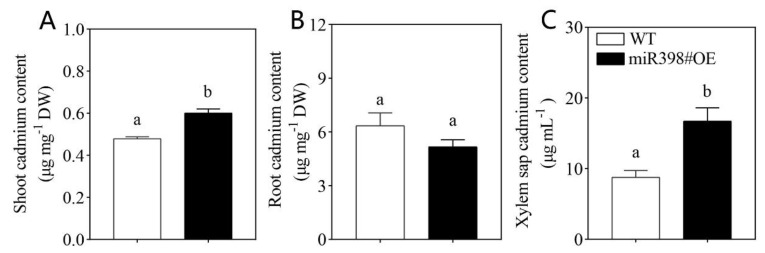
Cadmium contents in the shoot (**A**), root (**B**), and xylem sap (**C**) in wild type (WT) and transgenic line overexpressing *MIR398* (miR398#OE) under cadmium stress. Tomato seedlings (25 d old) of WT and miR398#OE were grown 100 μM Cd(NO_3_)_2_ for 9 d. The data are the mean values + SD of four individual replicates and different letters indicate significant differences (*p* < 0.05). DW, dry weight.

The concentrations of Cd in the shoot, root, and xylem sap of WT and miR398#OE were determined in this study (Figure 4). Although no obvious difference in root Cd concentration was observed between WT and miR398#OE, the concentration of Cd in the shoot and xylem sap was significantly higher in miR398#OE than that in WT by 25.3% and 90.5%, respectively (Figure 4).

**Figure 5:**
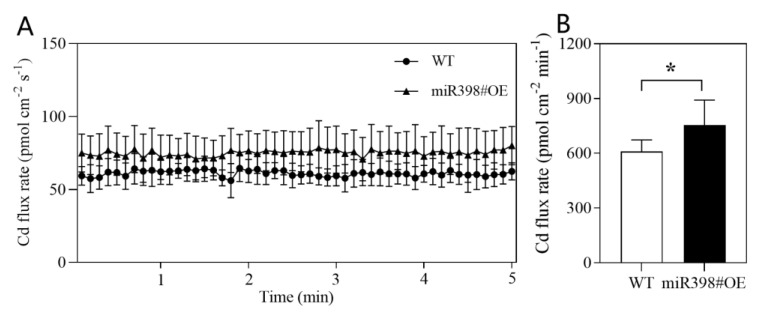
Cadmium flux rate in the root of wild type (WT) and transgenic line overexpressing *MIR398* (miR398#OE) under cadmium stress. Stable cadmium flux rate in tomato root in 5 min (**A**) and average cadmium flux rate based on time (**B**). Tomato seedlings (25 d old) of WT and miR398#OE were grown under 100 μM Cd(NO_3_)_2_ for 9 d. The data are the mean values ± or + SD of seven individual replicates. Asterisk indicates significant difference between WT and miR398#OE (*p* < 0.05).

Following the assay of Cd concentration in tomato, root net Cd influx rate was further measured using the non-invasive scanning ion-selected electrode technique (SIET), and a 5 min of stable Cd flux rate was recorded (Figure 5A). After calculating the net Cd influx rate based on time, the results showed overexpression of *MIR398* significantly increased net Cd influx rate by 23.6% (Figure 5B), which was in line with the enhanced Cd accumulation in tomato shoot of miR398#OE (Figure 4).

**Figure 6:**
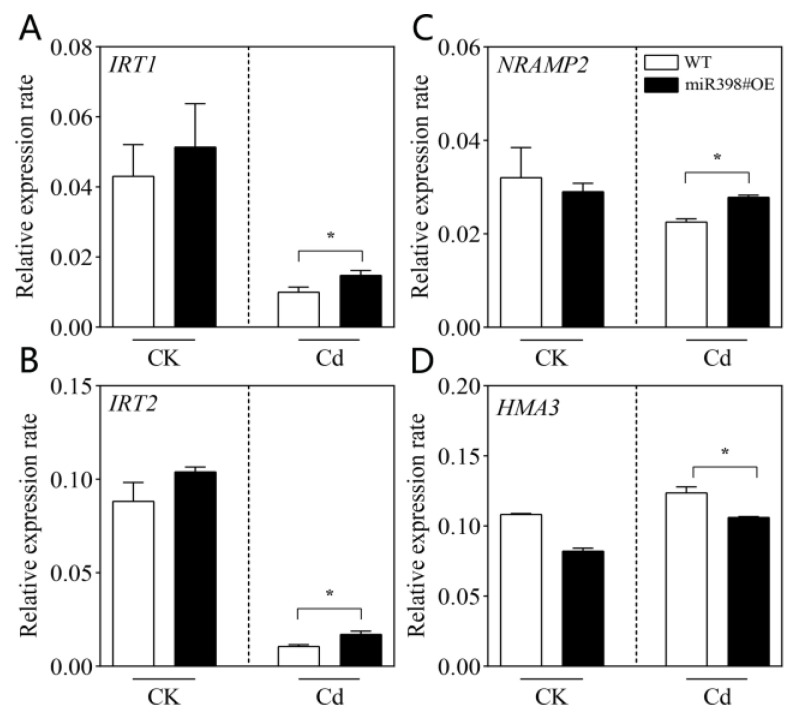
Relative expression rates of *IRT1* (**A**), *IRT2* (**B**), *NRAMP2* (**C**), and *HMA3* (**D**) in the root of wild type (WT) and transgenic line overexpressing *MIR398* (miR398#OE) under cadmium stress. Tomato seedlings (25 d old) of WT and miR398#OE were grown under control (CK) and 100 μM Cd(NO_3_)_2_ (Cd) for 9 d. The data are the mean values + SD of four individual replicates. Asterisks indicate significant difference between WT and miR398#OE (*p* < 0.05).

In addition, the expression of genes concerning Cd uptake (*IRT1*, *IRT2*, *NRAMP2*) and compartmentalization (*HMA3*) in the root of WT and miR398#OE was evaluated (Figure 6). The results showed that the expression of *IRT1*, *IRT2*, and *NRAMP2* was significantly up-regulated in miR398#OE root in contrast with that in WT (Figure 6A–C). Furthermore, the expression of *HMA3* was significantly down-regulated in the root of miR398#OE in comparison with WT (Figure 6D). The enhanced expression of Cd uptake genes and decreased expression of Cd compartmentalization genes could be responsible for the improved Cd concentration and net Cd influx rate in miR398#OE (Figure 4 and Figure 5).

## 3. Discussion

MicroRNAs (miRNAs) play key roles in regulating plant growth, development, and stress response [39,40]. As an important member of miRNAs in plants, microRNA398 (miR398) is involved in the acclimation to kinds of biotic and abiotic stresses, while the miR398-*CSD* module has been shown to play a central role in the regulatory network of miR398 in plants under stressful conditions [25].

In this study, our results showed the expression of miR398 was down-regulated in tomato under Cd exposure (Figure 1A), which is in line with the results in *Arabidopsis* under Cu stress [25]. Along with the decrease in miR398 expression, an increase in *CSD1* expression and SOD activity was observed in both the shoot and root in tomato under Cd stress (Figure 1B,D). However, the expression of *CSD2* was not changed by the down-expression of miR398 as *CSD1* in tomato under Cd exposure (Figure 1C). In plants, miR398 is able to modulate the activity of SOD by negatively regulating the expression of its target genes including *CSD1* and/or *CSD2* [21,25], while sequence match analysis showed the match between miR398 and *CSD1* was tighter than that between miR398 and *CSD2* [35]. In this study, our results indicated that *CSD1* but not *CSD2* is involved in the miR398-induced regulation of SOD in response to Cd toxicity in tomato. In addition, it should be noted that the down-regulated miR398 and up-regulated *CSD1* and SOD were found at 3 h after Cd treatment in the root but at 6 h after treatment of Cd in the shoot (Figure 1). In plants, the toxicity of Cd in plants is mainly based on Cd uptake and accumulation. Therefore, it is not surprising that the root is earlier in suffering from Cd toxicity than the shoot under Cd exposure [41,42], which was also proved by the earlier accumulation of H_2_O_2_ in the root (3 h) than the shoot (6 h) in this study. Overall, these results clearly indicate the down-expression of miR398 and its successive regulation on *CSD1* and SOD was in response to Cd accumulation and toxicity in tomato.

In plants under stressful conditions, the response of miR398 varies among stress types and plant species [21], implying that the distinct roles of miR398 in stress adaption. Therefore, to investigate the function of miR398 in Cd stress tolerance in tomato, a transgenic line overexpressing *MIR398* (miR398#OE) was further used in this study. Consistent with previous reports [43,44], Cd stress induced toxicity symptoms including growth inhibition, chlorophyll degradation, ROS accumulation, and membrane peroxidation in both wild type (WT, cv. Microtom) and miR398#OE. However, compared with WT, Cd stress induced more significant growth inhibition and oxidative damage in miR398#OE (Figure 2 and Figure 3). The results are consistent with that in *Arabidopsis* under Cu stress and high light stress [25], and in tomato under salt stress [35], which confirmed that the down-regulation of miR398 played a protective role in tomato against Cd stress.

However, it should be noted although Cd stress caused severe oxidative damage in both the shoot and root of tomato, overexpression of *MIR398* aggregated Cd-induced oxidative damage in the shoot but not in the root (Figure 3). Therefore, the activity of antioxidant enzymes in the shoot and root of miR398#OE and WT under Cd exposure was measured (Table 1). In the shoot of tomato, Cd stress induced significant decrease in SOD, CAT, and APX, and increase in GPOD and DHAR, while the Cd-induced disorder of antioxidant enzymes was further enhanced by *MIR398* overexpression (except DHAR) (Table 1). The results were in line with the enhanced oxidative damage in the shoot of miR398#OE (Figure 3). In the root, although Cd stress caused increase in APX, GPOD, GR, and DHAR, and decrease in SOD in WT, no aggregative effect of *MIR398* overexpression on these enzymes and oxidative damage (except H_2_O_2_) was found in this study. The regulation of miR398 on SOD under stress has been repeatedly documented in previous research [45]. In line with previous research concerning miR398 and the antioxidant system under biotic and abiotic stresses [35,36,37], our results indicate that, besides SOD, the miR398-*CSD1* module could also modulate the activity of other antioxidant enzymes in tomato under Cd stress, which could be based on the systematic cooperation of different kinds of enzymes in plant antioxidant defense [46,47,48].

Given the fact that the toxicity of Cd is based on Cd accumulation in plant tissue, which is mediated by root uptake and translocation of Cd [4], the concentration of Cd in tomato and Cd uptake flux rate was determined in this study. The content of Cd in the shoot and xylem sap was significantly higher in miR398#OE under Cd stress than that in WT, while no significant difference in root Cd concentration between miR398#OE and WT was observed (Figure 4). Moreover, the results of non-invasive scanning ion-selected electrode technique (SIET) showed that the net Cd influx rate was significantly higher in the root of miR398#OE than that in WT (Figure 5). By comparing the activity of antioxidant enzyme and Cd concentration in WT and miR398#OE under Cd toxicity, it can be concluded that the protective regulation effects of miR398 on antioxidant enzymes and Cd concentration were mainly observed in the shoot but not root of tomato. When subject to Cd stress, the shoot is more sensitive to Cd toxicity than the root [43,49]. Considering the important roles of the shoot in plant growth (e.g., photosynthesis), the protective effects of miR398 in the shoot would be more efficient in promoting Cd tolerance in tomato.

In plants, different genes including *IRT1*, *IRT2*, and *NRAMP2* have been shown to be involved in the process of root Cd uptake [41], while *HMA3* is responsible for vacuole compartmentalization, thereby influencing Cd translocation [50]. In this study, our results showed that overexpression of *MIR398* increased the expression of *IRT1*, *IRT2*, and *NRAMP2* (Figure 6A–C), and decreased the expression of *HMA3* (Figure 6D), which could be responsible for the enhanced Cd uptake and translocation in miR398#OE under Cd stress in comparison with WT. In the initial hypothesis, we have assumed that miR398 could influence the ability of ROS scavenge in plants under Cd stress, and successively influence affect membrane peroxidation and Cd transport in tomato under Cd exposure. However, overexpression of *MIR398* did not enhance Cd-induced oxidative damage in tomato root (Figure 3), implying that the regulation of miR398 on the activity of Cd transport and related genes is independent of membrane peroxidation. The mechanisms behind this still need further investigation.

## 4. Materials and Methods

### 4.1. Plant Growth and Treatment Application

Seeds of tomato (*Solanum lycopersicum* L.) were sterilized with distilled water at 55 °C for 10 min and then germinated on moist filter paper in dark at 28 °C for 2 d. After 20 d of growth on the substrate (Golden No.3, Jinhai, Hangzhou, China), each three uniform tomato seedlings were transplanted to a plastic bottle containing 1.2 L nutrient solution for another 3 d of growth. The nutrient solution (pH 6.2) was renewed each 3 d and contained 5 mM Ca(NO_3_)_2_, 5 mM KNO_3_, 2.5 mM KH_2_PO_4_, 2 mM MgSO_4_, 29.6 μM H_3_BO_3_, 10 μM MnSO_4_, 50 μM Fe-EDTA, 1.0 μM ZnSO_4_, 0.05 μM H_2_MoO_4_, and 0.95 μM CuSO_4_. The experiment was conducted in a growth chamber with a controlled environment: 70% relative humidity, 16/8 h, and 28/20 °C for light/dark period, respectively. After growth under the condition mentioned above, short- and long-time treatment experiment was conducted.

Experiment I: Tomato seedlings (cv. Microtom) were treated with or without 100 μM Cd(NO_3_)_2_ addition for 6 h, the expression of miR398, *CSD1*, *CSD2*, the activity of SOD, and the contents of superoxide anion (O_2_^−^) and H_2_O_2_ in tomato shoot and root were measured.

Experiment II: Tomato seedlings of Microtom (wild type, WT) and transgenic line overexpressing miR398 (miR398#OE) obtained from our previous work [35] were grown under control and 100 μM Cd(NO_3_)_2_ addition for 9 d. Then, tomato growth, oxidative damage, antioxidant enzyme activity, Cd concentration, net Cd flux rate, and the expression of genes concerning Cd uptake was evaluated.

### 4.2. Tomato Growth and Chlorophyll Concentration

Tomato seedlings were harvested with the root and shoot separated, and then fresh weight, shoot height, and root length were measured. For chlorophyll assay, leaf samples were homogenized with alcohol solution (95%, *v*/*v*), and the content of chlorophyll was determined by measuring the absorbance of the supernatant at 665, 649, and 470 nm with an UV/Vis spectrophotometer (UV-2600, Shimadzu, Kyoto, Japan) according to Porra et al. [51].

### 4.3. Oxidative Damage and ROS Accumulation

To evaluate the oxidative damage caused by Cd stress, nitroblue tetrazolium (NBT) and diaminobenzidine (DAB) staining were used for the visualization of superoxide anion (O_2_^−^) and H_2_O_2_ in tomato shoot samples according to the methods described by Jabs et al. [52] and Vanacker et al. [53], respectively. The contents of malonaldehyde (MDA), O_2_^−^, and H_2_O_2_ in the shoot and root of tomato under different treatments were measured using the trichloroacetic acid–thiobarbituric acid (TCA–TBA) method [54], hydroxylamine oxidation method [55], and potassium iodide method [56], respectively.

### 4.4. Antioxidant Enzyme Activity

Fresh tomato samples were homogenized with 1.5 mL phosphate butter (50 mM, pH 7.8) containing 0.2 mM ethylenediamine tetraacetic acid and 2% (*w*/*v*) polyvinyl pyrrolidone, and 1 mL supernatant was collected for the detection of soluble protein and antioxidant enzyme activity after centrifugation at 10,000 rpm for 10 min at 4 °C [57]. Superoxide dismutase (SOD) activity was determined with the NBT method and one unit (U) activity of SOD was defined as 50% inhibition of the photochemical reduction of NBT [58]. Catalase (CAT) activity was determined based on the extinction rate of H_2_O_2_ according to Cakmak and Maschner [59]. Ascorbate peroxidase (APX) activity was determined by ascorbate oxidation according to Nakano and Asada [60]. Guaiacol peroxidase (GPOD) activity assay was conducted with the method described by Egley et al. [61]. Dehydroascorbate reductase (DHAR) activity was calculated by the formation of ascorbate according to Mano et al. [62]. Glutathione reductase (GR) activity was defined by nicotinamide adenine dinucleotide phosphate oxidation according to Foyer and Halliwell [63].

### 4.5. Cd Concentration

Tomato shoot and root samples were oven-dried at 80 °C for 96 h, and then digested with 5 mL nitric acid and 1 mL hydrogen peroxide at 180 °C on a thermal digestion system (SH230N, Sineo, Shanghai, China). Xylem sap was collected according to Yan et al. [64] by cutting the stem off 2 cm above the root base. Cd concentrations were analyzed with inductively coupled plasma-mass spectrometry (ICP-MS, NexION 300X, Perkin Elmer, Waltham, MA, USA).

### 4.6. Net Cd Influx Rate

After 9 d of treatment, root samples (about 1.5 cm) were cut off above the root apex for Cd flux determination using non-invasive scanning ion-selected electrode technique (SIET, BIO-001A, Younger, Amherst, MA, USA) according to Yan et al. [65]. Briefly, root samples were placed in plastic dishes containing test buffer for about 1 h, and the electric potential was measured at about 4 μm above the root surface with a microelectrode for about 15 min. Then, the change in electric potential was converted into Cd flux rate according to Cd standard curves based on 50 and 500 μM Cd(NO_3_)_2_ (about 28–32 mv per decade) with Mageflux (Xuyue, China, http://xuyue.net/mageflux, accessed on 6 May 2015). The test buffer (pH = 5.8) contains 0.1 mM KCl, 0.3 mM MES, and 0.1 mM Cd(NO_3_)_2_, and the electrode was filled with Cd selected cocktail (about 50 μm) and 100 mM KCl solution (about 1 cm).

### 4.7. Gene Expression

Total RNA was extracted from frozen tomato samples (approximately 100 mg) using Trizol reagent (Invitrogen, Thermo Fisher, Waltham, MA, USA). After the measurement of A_260/280_ and A_260/230_ to check the purity and integrity, miRNA and RNA were converted to cDNA with a miRNA first-strand cDNA synthesis kit (by stem-loop) (Vazyme, Nanjing, China) and HiScript III first-strand cDNA synthesis kit (Vazyme, Nanjing, China), respectively. Quantitative real-time PCR was operated on a quantitative PCR system (qTower3, Analytik jena, Jena, Germany) using chamQ SYBR qPCR master mix (Vazyme, Nanjing, China) following the instruction. The relative expression rate was calculated using the ΔΔCt method. *U6* was for miR398, while *Actin* was used as the internal reference for *CSD1*, *CSD2*, *IRT1*, *IRT2*, *NRAMP2*, and *HMA3*. The sequence of primers used in this study were listed in supplementary Table S1.

### 4.8. Statistical Analysis

Statistical analysis was performed with Excel software (2019 version, Microsoft, Redmond, WA, USA) and data were compared using one-way or two-way analysis of variance (ANOVA). The significant difference was defined at the 0.05 probability level.

## 5. Conclusions

**Figure 7:**
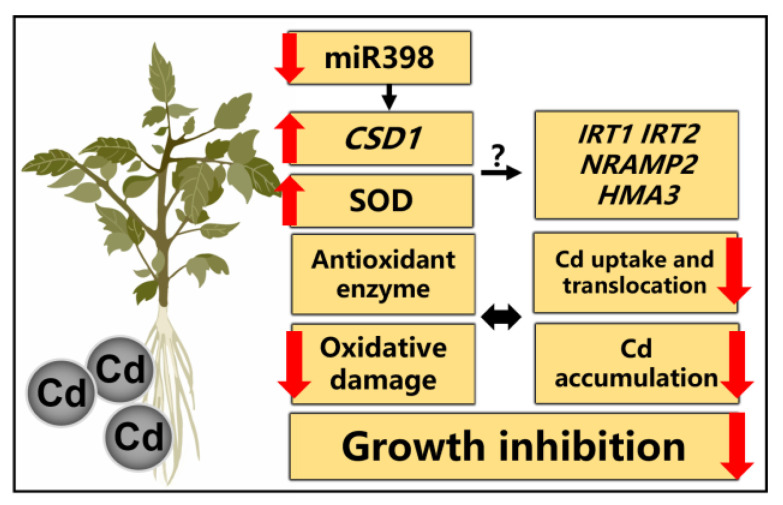
A schematic model of the role of miR398 in Cd stress acclimation in tomato.

In conclusion, our results show the down-regulation of miR398 plays a protective role in tomato against Cd stress through modulating antioxidant system and Cd uptake and translocation, thereby alleviating oxidative damage and promoting plant growth under Cd contamination (Figure 7). The results of this study would expand our knowledge concerning miRNA and metal stress tolerance in plants, which would also provide new insight for breeding and Cd pollution remediation.

## Figures and Tables

**Table 1:** Effects of cadmium stress on the activities of antioxidant enzymes in tomato shoot and root of wild type (WT) and transgenic line overexpressing *MIR398* (miR398#OE).

Treatment			SOD	CAT	APX	GPOD	GR	DHAR
Shoot	CK	WT	16.67 ± 0.02 A	27.60 ± 0.03 A	2.76 ± 0.12 A	30.72 ± 2.96 C	2.56 ± 0.53 A	71.98 ± 4.28 B
		miR398#OE	12.98 ± 0.16 B	27.56 ± 4.28 A	2.14 ± 0.07 B	38.12 ± 1.72 C	1.83 ± 0.30 AB	84.88 ± 3.30 B
	Cd	WT	12.23 ± 0.50 B	13.86 ± 1.12 B	2.04 ± 0.08 B	123.02 ± 11.67 B	2.05 ± 0.07 AB	143.95 ± 13.11 A
		miR398#OE	10.14 ± 0.87 C	3.67 ± 0.49 C	1.15 ± 0.06 C	159.33 ± 18.75 A	1.45 ± 0.22 B	145.72 ± 26.01 A
Root	CK	WT	50.36 ± 2.02 a	8.22 ± 1.60 a	3.82 ± 0.35 c	134.57 ± 9.03 c	5.66 ± 0.50 a	25.71 ± 1.69 b
		miR398#OE	40.01 ± 3.69 b	5.03 ± 0.40 b	4.94 ± 0.30 b	149.57 ± 6.66 c	3.15 ± 0.03 b	23.72 ± 1.78 b
	Cd	WT	23.04 ± 0.74 c	7.27 ± 0.62 a	6.73 ± 0.60 a	391.12 ± 6.88 a	2.99 ± 0.58 b	38.19 ± 2.37 a
		miR398#OE	26.90 ± 2.57 c	2.70 ± 0.40 c	7.32 ± 0.29 a	340.71 ± 4.70 b	3.11 ± 0.20 b	42.22 ± 0.88 a

Note: Tomato seedlings (25 d old) were grown under control (CK) and 100 μM Cd(NO_3_)_2_ (Cd) treatment for 9 d. The data are the mean values ± SD of four individual replicates and different letters show significant differences (*p* < 0.05). One unit (U) activity of SOD is defined as 50% inhibition of the photochemical reduction of nitroblue tetrazolium. SOD, superoxide dismutase, U mg^−1^ Pro; CAT, catalase, mM H_2_O_2_ mg^−1^ Pro min^−1^; APX, ascorbate peroxidase, mM ASA mg^−1^ Pro min^−1^; GPOD, guaiacol peroxidase, mM guaiacol mg^−1^ Pro min^−1^; GR, glutathione reductase, mM NADPH mg^−1^ Pro min^−1^; DHAR, dehydroascorbate reductase, mM DHA mg^−1^ Pro min^−1^; Pro, protein; ASA, ascorbate; DHA, dehydroascorbate.

## Data Availability

The data presented in this study are available in this manuscript.

## Supplementary Materials

The following supporting information can be downloaded at: https://www.mdpi.com/article/10.3390/ijms24031953/s1.
