# Supplementary material for: Sly-miR398 Participates in Cadmium Stress Acclimation by Regulating Antioxidant System and Cadmium Transport in Tomato (Solanum lycopersicum)
Source: Int J Mol Sci. 2023 Jan 19;24(3):1953. doi: 10.3390/ijms24031953 (PMC9915548; doi:10.3390/ijms24031953)
Supplement: Supplementary file 1 [file ijms-24-01953-s001.zip › ijms-2144804-supplementary.pdf]

Table S1 Primer sequences used in this

Gene	Forward Primer	Reverse Primer
U6	CATCCGATAAAATTGGAACGA	TTTGTGCGTGTCATCCTTGCG
miR398	GGCGGTGTGTTCTCAGGTCA	GTGCAGGGTCCGAGGT
Actin	TTCAAAGGGCGAGTACGACG	ACTTGCCTAACAGCAGACCC
CSD1	GGTGTTAGTGGCACCATCCT	AGCACCATGCTCCTTACCAG
CSD2	ACATTGTTGCTGGTCCTAATGAGA	CCCAATAACGCCTCTTCCCA
IRT1	GTAGTAGAAGATTGTGGAGCAG	CTTAGGGCCGGAATAGAA
IRT2	TTGCTATGTTGTCCGCTAT	GCCTTCAACTCCGTCTTT
NRAMP2	ATGCCTCACAATGTCTTCTTGC	CCTAAACTACCAGCTTGCTCACT
HMA3	TCACTCTACAACTATCCCTACTG	GAACAAACCTTACGACCACT
